# Endoscopic band ligation alone and combined with clipping for colonic diverticular bleeding: Retrospective comparative study

**DOI:** 10.1055/a-2536-7884

**Published:** 2025-03-14

**Authors:** Noritaka Ozawa, Kenji Yamazaki, Nae Hasebe, Kazuki Yamauchi, Kaori Koide, Hiroyuki Murase, Saeka Hayashi, Takaaki Hino, Daiki Hirota, Atsushi Soga, Kiichi Otani, Naoya Masuda, Hiroki Taniguchi, Shogo Shimizu, Masahito Shimizu

**Affiliations:** 1 68266 Department of Gastroenterology, Gifu Prefectural General Medical Center, Gifu, Japan; 2 38225 Department of Gastroenterology/Internal Medicine, Gifu University School of Medicine Graduate School of Medicine, Gifu, Japan

**Keywords:** Endoscopy Lower GI Tract, Lower GI bleeding, Diagnosis and imaging (inc chromoendoscopy, NBI, iSCAN, FICE, CLE...), Polyps / adenomas / ...

## Abstract

Clipping alone or endoscopic band ligation (EBL) alone are the main endoscopic hemostatic methods for colonic diverticular bleeding (CDB). We have established a novel method combining EBL and clipping (EBL-C) for hemostasis of CDB (Endoscopy E-videos); this study evaluated its usefulness. From March 2019 to July 2024, we endoscopically treated 138 patients for CDB at our institution. We retrospectively compared two groups: those treated with EBL (n = 24) and those treated with EBL-C (n = 56). Risk factors for early rebleeding were also examined in the EBL-C group. The rate of early rebleeding (defined as rebleeding occurring within 30 days) was lower in the EBL-C group than in the EBL group, although this difference was only marginally non-significant (8.9% vs. 25.0%, *P* = 0.0776). Failure of neck formation was the only independent risk factor for rebleeding (adjusted odds ratio [OR] 0.076; 95% confidence interval [CI] 0.015–0.398; *P* = 0.0023). Frequency of neck formation was significantly higher in the EBL-C group (EBL-C: 89.3% vs. EBL: 66.7%, *P* = 0.0235). Undergoing EBL-C was the only independent factor contributing to successful development of neck formation (adjusted OR 7.01; 95%CI 1.41–34.8; *P* = 0.0095). Previous treatment of the same diverticulum, neck formation failure, and insufficient clipping were risk factors for early rebleeding. Using EBL-C for CDB may be more effective in preventing rebleeding than using EBL alone because it facilitates better ligation of the target diverticulum. Treatment of diverticula that are hard and difficult to manage with suction remains a challenge.

## Introduction

Rates of colonic diverticular bleeding (CDB) are increasing in Japan, with patients hospitalized for bloody stools accounting for approximately 60% of cases 1 . Although spontaneous hemostasis is common in this condition and mortality risk is low, the high rebleeding rate remains a clinical concern. Methods of endoscopic hemostasis include clipping, ligation, local injection, and cauterization. However, a recent large-scale Japanese cohort study (CODE BLUE-J Study) has found that, in real-world clinical practice, clipping (57.2%) and ligation (41.0%) are most commonly used 2 . Clipping can be either direct or achieved via a diverticulum closure 3 . Meanwhile, ligation is broadly divided into endoscopic band ligation (EBL) and endoscopic detachable snare ligation 4 . Some recent studies have suggested that EBL may be more effective than clipping at preventing both early (within 30 days) and late (30 days to 1 year) rebleeding 5 6 7 , especially in cases involving active bleeding and right colon bleeding 2 . Although several previous studies have compared the efficacy of either method alone, combined use of these methods has not been assessed. We have previously reported on combined use of EBL and clipping (EBL-C) for a bleeding diverticulum, which involves hemostasis by direct clipping with a repositionable clip, followed by aspiration and bowel inversion for each clip 8 . Since November 2021, our first-choice method of hemostasis has been EBL-C, except for patients at risk of perforation due to long-term steroid therapy or maintenance dialysis. In this study, we aimed to compare clinical outcomes of EBL-C with those of EBL.

## Patients and methods

### Patients and study design

From March 2019 to July 2024, we conducted a retrospective review of medical records from 138 patients with CDB definitively diagnosed with stigmata of recent hemorrhage who underwent endoscopic hemostasis procedures at our department. Procedures included clipping (n = 50), EBL (n = 24), EBL-C (n = 56), and over-the-scope clipping (OTSC) (n = 8). We analyzed 80 cases treated with ligation procedures (EBL or EBL-C). EBL and EBL-C involve band ligation, and patients with a risk of perforation associated with ligation (e.g., those on maintenance dialysis or long-term steroid therapy) were excluded. In addition, cases in which diverticulum inversion was technically challenging were excluded due to difficulty of performing ligation. These patients were primarily treated using the clipping method.

We investigated overall treatment outcomes in patients who underwent ligation procedures (EBL or EBL-C). Subsequently, we compared patient characteristics and treatment outcomes between the EBL-C and EBL groups. Patient characteristics were comparable between both groups, including age, sex, underlying conditions, and medication use (e.g., antiplatelet agents, nonsteroidal anti-inflammatory drugs) ( Table 1 ).

**Table:** **Table 1** Characteristics of patients treated with EBL or EBL-C.

	EBL (n = 24)	EBL-C (n = 56)	*P* value
Age ≥ 70 years, n (%)	17 (70.8)	40 (71.4)	1 ^¶^
Sex, male, n (%)	15 (62.5)	27 (48.2)	0.329 ^¶^
Hypertension, n (%)	16 (66.7)	38 (67.9)	1 ^¶^
Antiplatelet agent, n (%)	6 (25)	19 (33.9)	0.599 ^¶^
Anticoagulant, n (%)	2 (8.3)	6 (10.7)	0.266 ^¶^
DAPT, n (%)	1 (4.17)	8 (14.3)	0.266 ^¶^
NSAIDs, n (%)	5 (20.8)	8 (14.3)	0.516 ^¶^
Bowel preparation (use of PEG solution), n (%)	12 (50)	22 (39.3)	0.461 ^¶^
Extravasation on contrast-enhanced CT, n (%)	9/20 (45)	30/49 (61.2)	0.286 ^¶^
Recent hemostasis ^*^ (< 30 days) (definitive cases ^†^ + possible cases ^‡^ ) n (%)	2 (8.3)	3 (5.4)	0.633 ^¶^
Past hemostasis ^§^ (> 30 days) (definitive cases + possible cases) n (%)	3 (0)	11 (3.6)	1 ^¶^
Location, left-side colon, n (%)	9 (26.8)	15 (36)	0.426 ^¶^
Use of long attachment cap, n (%)	22 (91.7)	53 (94.6)	0.633 ^¶^
^*^ Recent hemostasis is defined as hemostasis to the same diverticulum within 30 days. ^†^ Definitive cases: Treatment history of the same diverticulum was confirmed. ^‡^ Possible cases: Treatment history of the same diverticulum could not be ruled out. ^§^ Past hemostasis is defined as hemostasis to the same diverticulum more than 30 days prior. ^¶^ Fisher's exact test. CT, computed tomography; DAPT, dual antiplatelet therapy; EBL, endoscopic band ligation; EBL-C, endoscopic band ligation plus clipping; NSAID, nonsteroidal anti-inflammatory drug; PEG, polyethylene glycol.			

### Methods and devices

Identifying the responsible diverticulum is crucial. Therefore, performing bowel preparation within 24 hours and conducting the examination using a long hood attachment are recommended 9 10 11 . In addition, the identification rate increases when contrast-enhanced computed tomography (CT) shows evidence of extravasation 12 13 . Therefore, our hospital prioritizes performing contrast-enhanced CT whenever CDB is suspected. We recommend endoscopic examination after bowel preparation with polyethylene glycol within 24 hours. However, when extravasation is clearly visible on contrast-enhanced CT scans or the patient is hemodynamically unstable, emergency endoscopy may be conducted without bowel preparation at the attending physician’s discretion.

We used a water-jet scope, fitted with a long attachment cap, a SureClip (Micro-Tech Co., Nanjing, China), and an EBL device (Sumitomo Bakelite Co., Ltd., Tokyo, Japan).

### EBL-C procedure

Endoscopic hemostasis of colonic diverticular bleeding by combining endoscopic band ligation (EBL) and clipping.Video 1

**Fig. 1:**
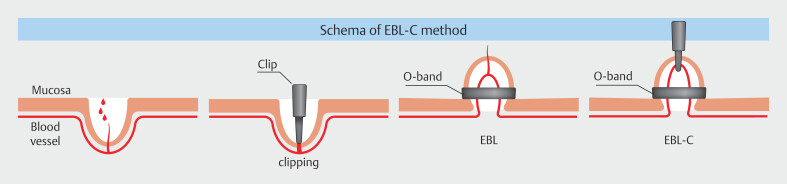
Schema of the EBL-C method.

EBL-C was conducted as follows ( Fig. 1 ) ( Video 1 ).

**Fig. 2:**
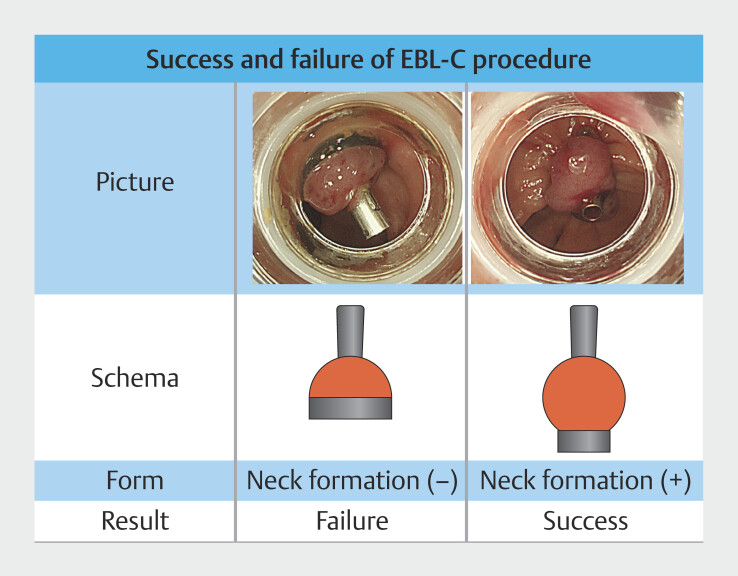
Success and failure of the EBL-C procedure.

1. Fit the long attachment cap. Once the bleeding diverticulum is identified, perform hemostasis by direct clipping (SureClip; Micro-Tech Co).
2. Withdraw the scope, fit the EBL device (Sumitomo Bakelite Co., Ltd.), and reinsert it. Suction the bleeding diverticulum containing the clip as far as possible, then release the O-ring. If suction and inversion are sufficient, ligation with the O-ring will have caused formation of a neck, indicating effective ligation ( Fig. 2 ).

### Statistical analysis

We used Fisher’s exact test for comparisons of categorical variables and a Mann–Whitney U test of the comparisons of continuous variables between groups. The association between endoscopic treatment and clinical outcomes was analyzed using univariate and multivariate logistic regression models. *P* < 0.05 was considered statistically significant. All statistical analyses were performed with EZR (Saitama Medical Center, Jichi Medical University, Saitama, Japan).

### Definition of terms

We focused on presence or absence of treatment history for the same diverticulum.

In cases of early rebleeding (within 30 days), identifying the same diverticulum was often possible based on evidence of prior procedures, such as residual clips or ulcers formed by EBL.

Conversely, identifying the same diverticulum after a longer period is often more challenging, but anatomical landmarks like the appendix or Bauhin's valve, as well as scarring, could aid determination. In addition, contrast-enhanced CT or endoscopy sometimes confirmed bleeding at a different site, ruling out involvement of the same diverticulum. Thus, we distinguished between definitive cases, in which treatment history of the same diverticulum was confirmed, and possible cases, in which involvement of the same diverticulum could not be ruled out.

## Results

Among patients who underwent ligation procedures (EBL or EBL-C) (n = 80), 11 cases (13.6%) experienced early rebleeding (within 30 days). In multivariate analysis of risk factors for early rebleeding, failure of neck formation was the only independent risk factor for rebleeding (adjusted odds ratio [OR] 0.076; 95% confidence interval [CI] 0.015–0.398; *P* = 0.0023) ( Table 2 ). Neck formation was achieved in 66 cases (82.5%) and undergoing EBL-C was the only independent factor contributing to successful development of neck formation (adjusted OR 7.01; 95% CI 1.41–34.8; *P* = 0.0095) ( Table 3 ).

**Table:** **Table 2** Early rebleeding risks after ligation therapy on logistic regression analyses.

	Early rebleeding (n = 11)	No rebleeding (n = 69)	Crude OR (95%CI)	*P* value	Adjusted OR (95%CI)	*P* value
Age ≥ 70 years, n (%)	9 (81.8)	48(69.6)	1.97 (0.39–9.91)	0.411		
Sex, female, n (%)	3 (27.3)	3(27.3)	2.75 (0.67–11.2)	0.16	1.92 (0.31–11.7)	0.48
Hypertension, n (%)	9 (81.8)	45(65.2)	2.4 (0.48–12.0)	0.287		
Diabetes, n (%)	1 (9.1)	14(20.1)	0.393 (0.04–3.33)	0.392		
Antiplatelet agent, n (%)	3 (27.3)	22(31.9)	0.801 (0.19–3.32)	0.76		
Anticoagulant, n (%)	2 (18.2)	6(8.7)	2.38 (0.41–13.4)	0.342		
DAPT, n (%)	1 (9.1)	8(11.6)	0.763 (0.09–6.77)	0.808		
NSAIDs, n (%)	3 (27.3)	10(14.5)	2.21 (0.5–9.78)	0.295		
Bowel preparation, n (%)	3 (27.3)	31(44.9)	0.46 (0.11–1.88)	0.28		
Recent hemostasis (< 30 days) (definitive + possible), n (%)	2 (18.2)	3(4.3)	4.89 (0.72–33.3)	0.105	4.22 (0.25–72.7)	0.322
Past hemostasis (> 30 days) (definitive + possible), n (%)	2 (18.2)	12(17.4)	1.06 (0.2–5.52)	0.949		
Location, left-side colon, n (%)	5 (45.5)	19(27.5)	2.19 (0.59–8.04)	0.236		
Use of long attachment cap, n (%)	9 (81.8)	66(95.7)	4.89 (0.72–33.3)	0.105	1.93 (0.17–22.4)	0.597
Neck formation (+), n (%)	4 (36.4)	62(89.9)	0.0645 (0.015–0.277)	0.000225	0.076 (0.015–0.398)	**0.00227**
EBL-C, n (%)	5 (45.5)	51(73.9)	0.294 (0.079–1.08)	0.0656	0.59 (0.12–2.82)	0.509
CI, confidence interval; DAPT, dual antiplatelet therapy; EBL-C, endoscopic band ligation plus clipping; NSAID, nonsteroidal anti-inflammatory drug; OR, odds ratio.						

**Table:** **Table 3** Factors associated with neck formation after ligation therapy on logistic regression analyses.

	Neck formation (+) (n = 66)	Neck formation (-) (n = 14)	Crude OR (95%CI)	*P* value	Adjusted OR (95%CI)	*P* value
Age ≥ 70 years, n (%)	45 (68.1)	12 (85.7)	0.357 (0.073–1.74)	0.203	0.297 (0.039–2.24)	0.238
Sex, female, n (%)	35 (53.0)	3 (21.4)	0.242 (0.062–0.95)	0.0414	0.3 (0.048–1.86)	0.195
Hypertension, n (%)	41 (62.1)	13 (92.9)	0.126 (0.0156–1.02)	0.0526	0.0682 (0.0031–1.49)	0.0879
Diabetes, n (%)	13 (19.7)	2 (14.3)	1.47 (0.29–7.40)	0.639		
Antiplatelet agent, n (%)	18 (27.3)	7 (50)	0.375 (0.115–1.22)	0.103	0.794 (0.14–4.55)	0.796
Anticoagulant, n (%)	5 (7.6)	3 (21.4)	0.301 (0.063–1.44)	0.133	0.183 (0.022–1.51)	0.114
DAPT, n (%)	8 (12.1)	1 (7.1)	1.79 (0.206–15.6)	0.597		
NSAIDs, n (%)	11 (16.7)	2 (14.3)	1.2 (0.24–6.13)	0.827		
Bowel preparation, n (%)	29 (43.9)	5 (35.7)	1.41 (0.43–4.67)	0.573		
Recent hemostasis (< 30 days) (definitive + possible), n (%)	2 (3.0)	3 (21.4)	0.115 (0.017–0.77)	0.0254	0.126 (0.0086–1.85)	0.131
Past hemostasis (> 30 days) (definitive + possible), n (%)	13 (19.7)	1 (7.1)	3.19 (0.38–26.6)	0.284		
Location, left-side colon, n (%)	20 (30.3)	4 (28.6)	1.09 (0.304–3.88)	0.898		
Use of long attachment cap, n (%)	3 (4.5)	2 (14.3)	0.286 (0.043–1.90)	0.195	0.394 (0.0387–4.01)	0.431
EBL-C, n (%)	50 (75.6)	6 (42.9)	4.17 (1.26–13.8)	0.0196	7.01 (1.41–34.8)	**0.0095**
DAPT, dual antiplatelet therapy; EBL-C, endoscopic band ligation plus clipping; NSAID, nonsteroidal anti-inflammatory drug; OR, odds ratio.						

The early rebleeding rate (rebleeding within 30 days) was lower in the EBL-C group than in the EBL group, although this difference was only marginally non-significant (8.9% vs. 25.0%, *P* = 0.0776). Over the 1-year follow-up period, the late rebleeding rate (rebleeding between 30 days and 1 year) was also lower in the EBL-C group than in the EBL group, but the difference was non-significant (2.4% vs. 8.3%, *P* = 0.0549). Conversely, the frequency of neck formation, considered a factor potentially reducing rebleeding risk, was significantly higher in the EBL-C group (EBL-C: 89.3% vs. EBL: 66.7%, *P* = 0.0235). There were no significant differences in use of red blood cell transfusion, treatment time, or hospitalization duration ( Table 4 ).

**Table:** **Table 4** Outcomes of patients treated with EBL or EBL-C.

	EBL (n = 24)	EBL-C (n = 56)	*P* value
Neck formation, n (%)	16 (66.7)	50 (89.3)	**0.0235** ^¶^
Early rebleeding*, n (%)	6 (25)	5 (8.9)	0.0776 ^¶^
Late rebleeding within 2 years ^†^ , n (%)	2/24 (8.3)	1/27 (3.7)	0.187 ^¶^
Late rebleeding within 1 year ^†^ , n (%)	2/24 (8.3)	1/41 (2.4)	0.0549 ^¶^
Time to hemostasis ^‡^ , median (range), min	20 (7–46)	21 (7–47)	0.871 ^††^
EBL procedure time ^§^ , median (range), min	15 (7–25)	15 (4–42)	0.617 ^††^
Transfusion, n (%)	11 (45.8)	29 (51.8)	0.808 ^¶^
Length of hospital stay after hemostasis, median (range), day	6 (4–23)	5 (1–14)	0.208 ^††^
Complications, n (%)	0 (0)	0 (0)	NA
*Early rebleeding: rebleeding that occurs within 30 days following the initial hemostasis. ^†^ Late rebleeding: rebleeding that occurs between 30 days and 1 year (or 2 years) following initial hemostasis. ^‡^ Time to hemostasis was defined as time to hemostasis complete after identifying the bleeding site. ^§^ EBL procedure time was defined as time to hemostasis complete after initial clipping. ^¶^ Fisher's exact test. ^††^ Mann–Whitney U test. EBL, endoscopic band ligation; EBL-C, endoscopic band ligation plus clipping.			

Rebleeding risk was examined in the EBL-C group. A definitive history of treatment for the same diverticulum over 30 days prior was significantly associated with higher risk of rebleeding. No other background factors were found to be significant ( Table 5 ).

**Table:** **Table 5** Patient-related risk factors for early rebleeding after EBL-C on Fisher‘s exact test.

	Early rebleeding (n = 5)	No rebleeding (n = 51)	*P* value*
Age ≥ 70 years, n (%)	4 (80)	36 (70.6)	1
Sex, male, n (%)	3 (60)	20 (39.2)	1
Hypertension, n (%)	5 (100)	33 (64.7)	0.164
Diabetes, n (%)	0 (0)	12 (23.5)	0.574
Antiplatelet agent, n (%)	2 (40)	17 (33.3)	1
Anticoagulant, n (%)	2 (40)	4 (7.8)	0.0836
DAPT, n (%)	1 (20)	7 (13.7)	0.522
NSAIDs, n (%)	1 (20)	7 (13.7)	0.552
Bowel preparation, n (%)	1 (20)	21 (41.2)	0.638
Early colonoscopy, n (%)	3 (60)	36 (70.6)	0.634
Extravasation on contrast-enhanced CT, n (%)	4/5 (80)	26/44 (59.1)	0.636
Use of long attachment cap, n (%)	4 (80)	49 (96.1)	0.249
Recent hemostasis (definitive), n (%)	0 (0)	3 (5.9)	1
Recent hemostasis (definitive + possible), n (%)	0 (0)	3 (5.9)	1
Past hemostasis (definitive), n (%)	2 (40)	0 (0)	**0.00649**
Past hemostasis (definitive + possible), n (%)	2 (40)	9 (17.6)	0.251
Location, right-side colon, n (%)	3 (60)	38 (74.5)	0.602
*Fisher’s exact text. CT, computed tomography; DAPT, dual antiplatelet therapy; EBL-C, endoscopic band ligation plus clipping; NSAID, nonsteroidal anti-inflammatory drug.			

Among procedure-related factors, rates of no neck formation and insufficient clipping were higher in the early rebleeding group than in the comparison group ( Table 6 ).

**Table:** **Table 6** Procedure-related risk factors for early rebleeding after EBL-C on Fisher’s exact test.

	Early rebleeding (n = 5)	No rebleeding (n = 51)	*P* value
Neck formation (-), n (%)	3 (60)	3 (5.9)	**0.00661** ^*^
Insufficient clipping ^)†^ , n (%)	3 (60)	4 (7.8)	**0.0112** ^*^
*Cases of insufficient hemostasis with initial clipping. ^†^ Fisher's exact test. EBL-C, endoscopic band ligation plus clipping.			

Among the EBL-C patients (n = 56), 44 (78.6%) successfully achieved both clip hemostasis and neck formation, with none experiencing early rebleeding (0/44). However, 11 patients (19.6%) had either unsuccessful clip hemostasis or neck formation, and early rebleeding occurred in four of them (36.4%). In addition, one patient (1.8%) had both unsuccessful clip hemostasis and neck formation and experienced early rebleeding. No complications occurred in either group.

Regarding rebleeding cases, rebleeding from the same diverticulum was observed in eight cases in the EBL group (early: 6, late: 2) and six in the EBL-C group (early: 5, late: 1). Methods of hemostasis for rebleeding EBL cases were as follows: for the six early rebleeding cases, one was treated with EBL-C, three with clipping, one achieved spontaneous hemostasis, and one was treated with OTSC. For the two late rebleeding cases, one was treated with EBL and one achieved spontaneous hemostasis. For rebleeding EBL-C cases, hemostasis methods were as follows: for the five early rebleeding cases, four were treated with OTSC and one with clipping. For the one late rebleeding case, EBL-C was performed. None of the five rebleeding cases treated with OTSC experienced rebleeding again.

In this study, there were seven definitive cases of treatment history in the same diverticulum.

There were five definitive cases of recent treatment history (within 30 days), including four treated with clipping and one with EBL. Among the clipping cases, the clips had dislodged in two cases and remained in place in two cases. The bands had dislodged, resulting in ulcer formation. There were two definitive cases of past treatment history (over 30 days prior), including one treated with EBL and one with EBL-C. In both definitive cases of past treatment history (over 30 days prior), clips and bands had dislodged, and although scarring was observed, the diverticulum itself remained. Both cases were treated with EBL-C, but early rebleeding occurred in both cases.

## Discussion

Unsuccessful neck formation was a risk factor for early rebleeding in ligation procedures. In EBL-C, which involves performing EBL after placing a clip, the rate of neck formation was higher compared with EBL alone, suggesting a potential reduction in rebleeding risk.

EBL-C is a simple procedure with several advantages. First, it involves hemostasis at two different parts of the vessel using different methods: clipping the exposed part of the vessel and using EBL on the non-exposed part. This dual approach enhances the hemostatic force, potentially increasing the effectiveness of rebleeding prevention.

Conventional EBL involves marking the vicinity of the bleeding diverticulum, which may prove difficult to identify while placing the EBL device and reinserting the scope. In EBL-C, because the bleeding diverticulum itself is clipped, there is no chance that it will be misidentified or overlooked, and EBL can be conducted with confidence.

In addition, in conventional EBL, after the vicinity of the diverticulum is marked with a clip, it continues to bleed while the scope is being temporarily withdrawn to place the EBL device before reinserting the scope, which can cause hemodynamic fluctuations and obscure the visual field. In EBL-C, because the bleeding diverticulum itself is clipped to stop the bleeding, hemostasis is usually achieved, and thus, the operator can continue the procedure without feeling under pressure.

Depending on the location of the responsible diverticulum, obtaining a frontal view may be difficult. With conventional EBL, the surrounding mucosa may be aspirated, making it challenging to invert the diverticulum. By performing EBL after clipping, we have managed multiple cases in which the clip acted as an axis, making it easier to identify the diverticulum center and perform aspiration. These experiences formed the basis for developing the EBL-C method.

Some concerns involve risk that presence of a clip within the diverticulum in EBL-C might make it difficult to fully pull the diverticulum into the EBL device during suction. However, in this study, the EBL-C group showed a significantly higher rate of neck formation (EBL-C 89.3% vs. EBL 66.7%, *P* = 0.0235). In practice, we seldom had the impression during the procedure that the diverticulum could not be fully pulled into the device. This suggests that presence of the clip may support sufficient suction by aligning the axis of aspiration.

Although we used maximum suction strength, we have not experienced a case in which the clip was dislodged by this suction. This procedure may require use of clips with a strong gripping force, such as a SureClip. Evidence from comparative studies involving other clips is required.

EBL-C patients who developed rebleeding had insufficient clipping and unsuccessful neck formation. Early rebleeding did not occur in any patients without these characteristics, and their risk of rebleeding can be regarded as extremely low. Conversely, if either of these characteristics is present, risk of rebleeding is high, and additional preventive treatment should be considered. Furthermore, both patients with definitive histories of past hemostasis for the same diverticulum experienced rebleeding. Absence of neck formation and a history of past hemostasis for the same diverticulum may both reflect hardness of the diverticulum.

EBL is considered challenging to perform when the diverticulum is hard because it makes suction and inversion difficult. In such cases, OTSC (Ovesco Endoscopy, Tübingen, Germany) reportedly is effective 14 15 . In this study, we conducted hemostasis using OTSC for patients who developed early rebleeding after ligation procedures (either EBL or EBL-C), with good outcomes. Data on use of OTSC for CDB are scarce and OTSC is associated with risk of complications, high cost, and extended treatment time. Further studies are required to verify whether OTSC should be recommended for all patients with risk factors for rebleeding.

There are many reports evaluating risk of rebleeding based on patient characteristics and diverticular features before treatment 16 17 18 19 . However, few studies have assessed risk of rebleeding according to procedure-related factors, such as post-ligation morphology. The only report examining complete eversion as a risk factor for bleeding showed a trend toward lower frequency of complete eversion in the rebleeding group, although no statistically significant differences were observed 20 . If the rebleeding risk could be assessed immediately after the procedure, this would be significant for CDB treatment, which has a higher risk of rebleeding and hemodynamic instability compared with other conditions.

Limitations of this study include a retrospective single-center design and small sample size. In addition, this study may be affected by sampling bias because treatment methods varied across different time periods. To further prove its efficacy, it will be necessary to conduct multicenter, randomized, controlled trials and accumulate evidence.

## Conclusions

In conclusion, EBL-C for CDB may be more effective in preventing rebleeding than EBL alone because it facilitates better ligation of the target diverticulum.

## References

[1] Nagata N Kobayashi K Yamauchi A Identifying bleeding etiologies by endoscopy affected outcomes in 10,342 cases with hematochezia: CODE BLUE-J Study Am J Gastroenterol 2021 116 2222 2234 34388140 10.14309/ajg.0000000000001413 PMC8560163

[2] Kobayashi K Nagata N Furumoto Y Effectiveness and adverse events of endoscopic clipping versus band ligation for colonic diverticular hemorrhage: a large-scale multicenter cohort study Endoscopy 2022 54 735 744 10.1055/a-1705-0921 34820792 PMC9329063

[3] Kishino T Nagata N Kobayashi K Endoscopic direct clipping versus indirect clipping for colonic diverticular bleeding: A large multicenter cohort study United European Gastroenterol J 2022 10 93 103 10.1002/ueg2.12197 PMC8830273 35020977

[4] Akutsu D Narasaka T Wakayama M Endoscopic detachable snare ligation: A new treatment method for colonic diverticular hemorrhage Endoscopy 2015 47 1039 1042 10.1055/s-0034-1392204 26021310

[5] Nakano K Ishii N Ikeya T Comparison of long-term outcomes between endoscopic band ligation and endoscopic clipping for colonic diverticular hemorrhage Endosc Int Open 2015 3 E529 E533 26528513 10.1055/s-0034-1392510 PMC4612232

[6] Nagata N Ishii N Kaise M Long-term recurrent bleeding risk after endoscopic therapy for definitive colonic diverticular bleeding: band ligation versus clipping Gastrointest Endosc 2018 88 841 8.53E6 30036505 10.1016/j.gie.2018.07.018

[7] Tsuruoka N Takedomi H Sakata Y Recent trends in treatment for colonic diverticular bleeding in Japan Digestion 2020 101 12 17 10.1159/000504089 31722336

[8] Ozawa N Yamazaki K Koizumi H Novel method combining endoscopic band ligation and clipping for hemostasis of colonic diverticular bleeding Endoscopy 2023 55 E887 E888 10.1055/a-2109-1195 37442176 PMC10344617

[9] Niikura R Nagata N Aoki T Predictors for identification of stigmata of recent hemorrhage on colonic diverticula in lower gastrointestinal bleeding J Clin Gastroenterol 2015 49 e24 e30 10.1097/MCG.0000000000000140 24859714

[10] Pasha SF Shergill A Acosta RD The role of endoscopy in the patient with lower GI bleeding Gastrointest Endosc 2014 79 875 885 24703084 10.1016/j.gie.2013.10.039

[11] Kobayashi M Akiyama S Narasaka T Multicenter propensity score-matched analysis comparing short versus long cap-assisted colonoscopy for acute hematochezia JGH Open 2023 7 487 496 10.1002/jgh3.12936 37496816 PMC10366493

[12] Sugiyama T Hirata Y Kojima Y Efficacy of contrast-enhanced computed tomography for the treatment strategy of colonic diverticular bleeding Intern Med 2015 54 2961 2967 10.2169/internalmedicine.54.5097 26631877

[13] Nakatsu S Yasuda H Maehata T Urgent computed tomography for determining the optimal timing of colonoscopy in patients with acute lower gastrointestinal bleeding Intern Med 2015 54 553 558 25786443 10.2169/internalmedicine.54.2829

[14] Kawano K Takenaka M Kawano R Efficacy of over-the-scope clip method as a novel hemostatic therapy for colonic diverticular bleeding J Clin Med 2021 10 2891 10.3390/jcm10132891 34209655 PMC8268121

[15] Yamazaki K Maruta A Taniguchi H Endoscopic treatment of colonic diverticular bleeding with an over-the-scope clip after failure of endoscopic band ligation VideoGIE 2020 5 252 254 32529161 10.1016/j.vgie.2020.02.017 PMC7278022

[16] Yamauchi A Ishii N Yamada A Outcomes and recurrent bleeding risks of detachable snare and band ligation for colonic diverticular bleeding: a multicenter retrospective cohort study Gastrointest Endosc 2023 98 1 10.1016/j.gie.2023.02.014 36801460

[17] Yamauchi A Kou T Kishimoto T Risk factor analysis for early rebleeding after endoscopic treatment for colonic diverticular bleeding with stigmata of recent hemorrhage JGH Open 2021 5 573 579 34013057 10.1002/jgh3.12535 PMC8114991

[18] Nishikawa H Maruo T Tsumura T Risk factors associated with recurrent hemorrhage after the initial improvement of colonic diverticular bleeding Acta Gastroenterol Belg 2013 76 20 24 23650778

[19] Kawanishi K Kato J Kakimoto T Risk of colonic diverticular rebleeding according to endoscopic appearance Endosc Int Open 2018 6 E36 E42 10.1055/s-0043-122494 29340296 PMC5766334

[20] Ikeya T Ishii N Nakano K Risk factors for early rebleeding after endoscopic band ligation for colonic diverticular hemorrhage Endosc Int Open 2015 3 E523 E528 10.1055/s-0034-1392215 26528512 PMC4612237

